# Gallic acid oxidation products alter the formation pathway of insulin amyloid fibrils

**DOI:** 10.1038/s41598-020-70982-3

**Published:** 2020-09-02

**Authors:** Andrius Sakalauskas, Mantas Ziaunys, Vytautas Smirnovas

**Affiliations:** Life Sciences Center, Institute of Biotechnology, Vilnius University, Sauletekio al. 7, 10257 Vilnius, Lithuania

**Keywords:** Protein aggregation, Biophysical chemistry

## Abstract

Amyloidogenic protein assembly into insoluble fibrillar aggregates is linked with several neurodegenerative disorders, such as Alzheimer’s or Parkinson’s disease, affecting millions of people worldwide. The search for a potential anti-amyloid drug has led to the discovery of hundreds of compounds, none of which have passed all clinical trials. Gallic acid has been shown to both modulate factors leading to the onset of neurodegenerative disorders, as well as directly inhibit amyloid formation. However, the conditions under which this effect is seen could lead to oxidation of this polyphenol, likely changing its properties. Here we examine the effect of gallic acid and its oxidised form on the aggregation of a model amyloidogenic protein–insulin at low pH conditions. We show a vastly higher inhibitory potential of the oxidised form, as well as an alteration in the aggregation pathway, leading to the formation of a specific fibril conformation.

## Introduction

Protein aggregation into highly-structured, beta-sheet rich fibrils is associated with multiple neurodegenerative disorders, such as Alzheimer’s, Parkinson’s or prion diseases^1,2^. The prevalence of these diseases is of utmost concern, as there is currently no available drug or effective treatment^3^. As of right now, there are various potential antibodies, peptides, naturally occurring polyphenolic molecules and other compounds with either direct or indirect anti-amyloid activity^3–5^. However, a large portion of them are either in initial trials or do not work in vivo and most have not passed the third clinical trial stage^6^.

Tea extracts are known for their beneficial effects on health and a wide variety of compounds present in them have been shown to possess anti-amyloid properties, with one of them being gallic acid (GA)^7,8^. This simple polyphenol is considered to be able to not only mediate the factors leading to neurodegenerative diseases, such as oxidative stress or inflammation, but also to directly inhibit the formation of amyloid fibrils^9–22^.

In these experiments, one of the methods used to track the existence or formation of amyloids is an amyloidophilic dye molecule–thioflavin-T (ThT), which fluorescence emission intensity increases significantly when it binds to the beta-sheet grooves on the surface of amyloid fibrils^23^. In some cases, where GA or other polyphenolic compounds displayed a reduction in ThT fluorescence, additional examinations by alternative methods, such as transmission electron microscopy, revealed that such a fluorescence inhibition may be related to interference between the dye and inhibitor^24^. This could be the result of either fluorescence quenching due to molecule interactions or as an inner filter effect, because of the absorbance of ThT emissions by the inhibitor^25^. Despite this, multiple reports consider the reduction of ThT fluorescence as inhibition of aggregation^10–12,14,16,17,19,22,26^.

The effect of GA is also usually determined in neutral pH both in vitro and in vivo^11,12,16,17,19,20,26^. And it is known that it is capable of undergoing oxidation at neutral and higher pH^26–29^. One report demonstrated that when this process is carried out at pH 7.4, a *o*-quinone is produced and changes in the solution’s absorbance spectra can be observed^26^. In another instance, incubation of GA at neutral pH lead to the formation of a gallic acid dimer^30^. Oxidation at highly basic conditions was shown to yield ellagic acid^31^. The varying reports make it highly likely that when GA is incubated under neutral conditions, the product may be a mixture of two or more different compounds.

It was shown for other natural polyphenolic compounds, such as EGCG, that oxidation alters their anti-amyloid effects in a positive way^32,33^. The appearance of new oxidised GA forms could result in the modulation of different aggregation pathways, as well as have higher or lower aggregation inhibition or fibril disassembly potential. In addition, it was shown that during auto-oxidation, some of the reactions could lead to the formation of hydrogen peroxide^34^. This could, in turn, result in the hydroxylation of ThT and cause the previously mentioned reduction in fluorescence intensity^35^. All of these factors may lead to a false interpretation of the inhibitory potential of GA; therefore, its effect requires further analysis.

A commonly used model protein to examine anti-amyloid compound activity is insulin^36^. The majority of insulin aggregation studies are carried out at low pH conditions^14,37–40^, with some being conducted at neutral pH^41,42^. Depending on the acidity of the solution, insulin can exist as a monomer, dimer, tetramer or hexamer^43^. This greatly complicates matters when attempting to fit experimental data to an aggregation model, as it needs to account for the formation and existence of these non-monomeric assemblies, which can even directly affect the aggregation process^44^. It is known that insulin exists as a monomer in a 20% acetic acid solution^43^, which negates the need for the model to account for any oligomeric forms and it can be broken down into four basic steps, which include primary nucleation, fibril elongation, secondary nucleation on the surface of fibrils and fragmentation of aggregates^45^. We have also recently reported that when insulin is aggregated in a 20% acetic acid solution, it forms two distinct fibril conformations, based on the initial concentration of the protein^46^. Fibrils, formed at low protein concentration (further referred as low concentration fibrils or LCF) are short, dispersed and result in a low fluorescence intensity of fibril-bound-ThT, while fibrils formed at high protein concentration (high concentration fibrils or HCF) are longer, clumped together and result in a high fluorescence intensity of fibril-bound-ThT. This creates an additional opportunity to examine whether the effect of GA is dependent on the conformation of aggregates. Such a low pH solution should also prevent or minimize the oxidation of GA, which allows to explore its inhibitory effect without the appearance of any of its oxidised forms.

In this work we examine the effect of GA and how it modulates the mechanism of insulin amyloid aggregation. We show that its oxidation products have a significantly higher inhibitory effect on the formation of primary nuclei and favour a specific insulin fibril conformation.

## Methods

### Insulin sample preparation

Human recombinant insulin powder (Sigma-Aldrich cat. No. 91077C) was dissolved in a 20% acetic acid solution (prepared from 100% acetic acid; Carl-Roth, cat. No. 3738.1) containing 100 mM NaCl (Fisher cat. No. 10316943, purity > 99.5%), which is further referenced as the reaction solution, to a final concentration of 2 mM (11.6 mg/ml). Samples for unseeded aggregation kinetic measurements were prepared by diluting the 2 mM stock solution to a range of concentrations from 0.2 to 1.0 mM by using the reaction solution, as well as 10 mM ThT (Sigma-Aldrich, cat. No. T3516) and 10 mM gallic acid (TCI Chemicals, cat. No. G0011, purity > 98%) stock solutions (final ThT concentration was 100 µM in all cases, gallic acid concentration was in the range from 0 to 200 µM). For seeded aggregation, insulin fibrils, prepared from the 0.2 mM sample (which did not contain gallic acid), were sonicated for 10 min using Sonopuls 3,100 (Bandelin) ultrasonic homogenizer equipped with a MS73 tip (40% power, 30 s sonication/30 s rest intervals). The homogenized fibrils were then mixed with the insulin, gallic acid and ThT stock solutions to yield 0.2 and 1.0 mM unaggregated protein concentration samples containing 100 µM ThT, and a range of fibril concentrations (from 1 to 10^−6^% of total protein mass) with and without 200 µM gallic acid.

### Gallic acid solution preparation

Non-oxidised gallic acid (GA) stock solution was prepared by dissolving 10 mM gallic acid in the reaction solution. Oxidised gallic acid (GAO) stock solution was prepared by dissolving 10 mM gallic acid in a 100 mM sodium phosphate (Carl-Roth, cat. No. P030.3 and T879.2, purity > 99%) buffer (pH 7.4). Oxidation was achieved by incubating the solution at 37 °C for 15 days. Absorbance spectra of GA at the start and end of the reaction were scanned in the range from 250 to 800 nm to confirm changes to its structure (Fig. S1).

### Aggregation kinetics

Insulin aggregation kinetics were monitored in non-binding 96-well plates (sample volume was 100 µL) at 60 °C without agitation by measuring ThT fluorescence emission intensity (excitation wavelength—440 nm, emission—480 nm) through the bottom of the plate, using Synergy H4 Hybrid Multi-Mode (Biotek) microplate reader (readouts were taken every 10 min). For every condition 3 independent measurements were performed. In order to rule out any possible effect ThT may have on the aggregation measurements, the aggregation was simultaneously tracked by ThT fluorescence and sample optical density at 600 nm (Fig. S2).The ThT fluorescence intensity was normalized and the aggregation half-time (t_50_) values were calculated by applying a linear fit to the data points ranging from 40 to 60% of normalised intensity values and interpolating the time at which 50% of intensity is reached. The increase in t_50_ was used as a main hallmark of inhibition.

### Atomic force microscopy (AFM)

After kinetic measurements, the sample AFM images were scanned as previously described^46^. In short, 20 µL of each sample was deposited on freshly cleaved mica and incubated for 1 min. Then the samples were rinsed with MilliQ water and dried under airflow. AFM images were scanned using a Dimension Icon (Bruker) atomic force microscope. The 1,024 × 1,024 pixel resolution images were analysed using Gwyddion 2.55. Fibril length, height and width were determined by tracing parallel and perpendicular to each fibril’s axis.

### Fourier-transform infrared (FTIR) spectroscopy

Insulin fibrils were separated from solution by centrifugation at 10,000 g for 30 min and subsequently resuspended in 1 mL of D_2_O, the procedure was repeated 3 times. After the last centrifugation the fibrils were resuspended in 0.25 mL of D_2_O and sonicated for 1 min using a MS72 tip (with 50% power and constant sonication). FTIR spectra were recorded and analysed as previously described^46^.

### Analysis of aggregation kinetics

Experimental data fitting was done using rModeler (Ubicalc Software) as described previously^44^ and the model’s mathematical framework is provided as supplementary information. In short, a “classic” model comprised of four aggregation steps, including primary nucleation, elongation, secondary nucleation and fragmentation, was applied to fit the kinetic data. Three combined rate constants (primary nucleation-elongation, elongation-secondary nucleation and elongation-fragmentation)^44,47,48^ were obtained for every condition. Each set of kinetic constants is the average of 3 data set fits (insulin concentration is in micromoles; nuclei size is set to 2).

## Results

### Aggregation kinetics

**Figure 1:**
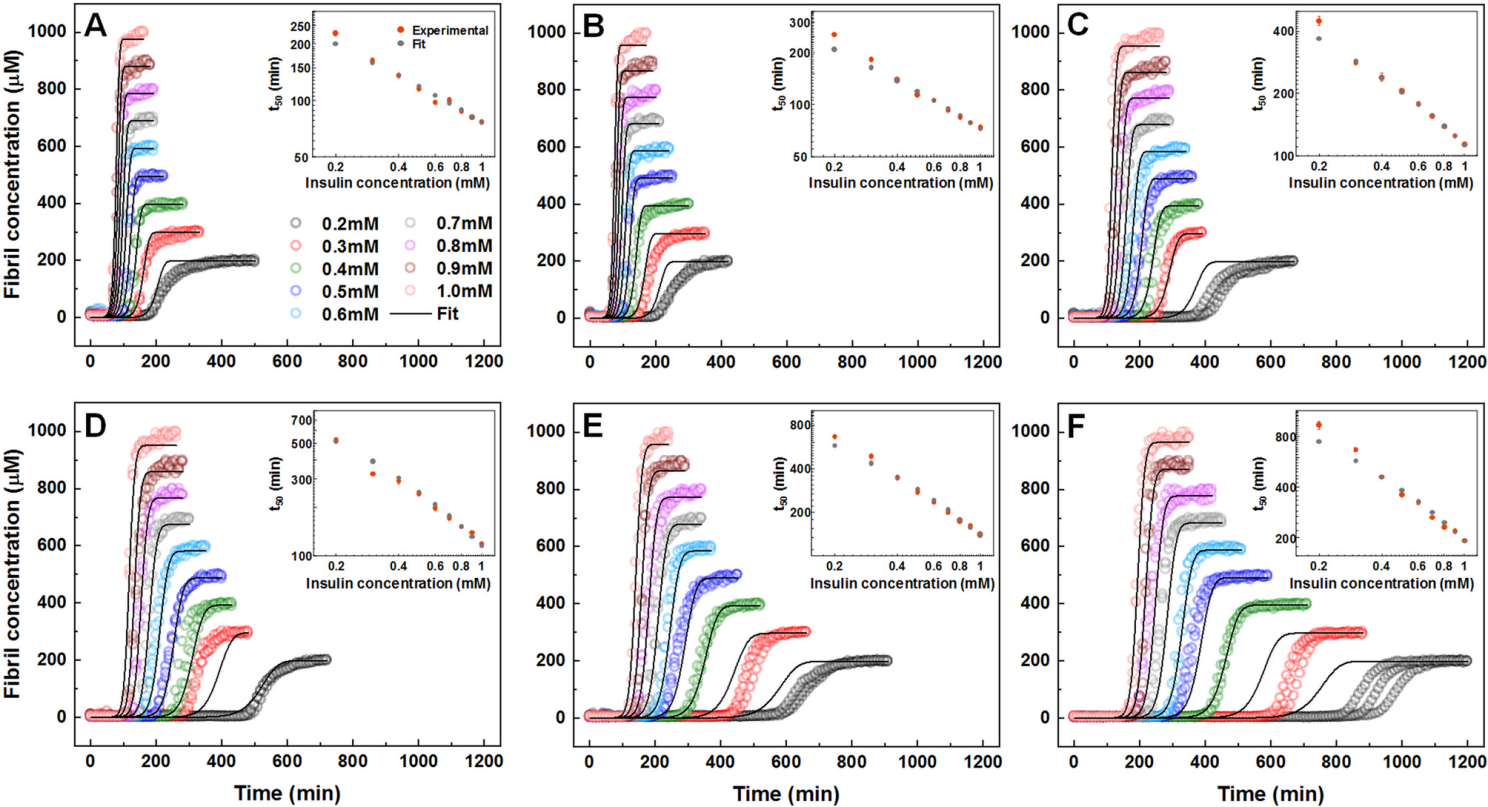
Unseeded aggregation kinetics of insulin without GA (**A**), with 200 µM GA (**B**), 25 µM GAO (**C**), 50 µM GAO (**D**), 100 µM GAO (**E**) and 200 µM GAO (**F**). For each condition, global-fitting was applied using a four-step aggregation model. Inserts show a comparison between t_50_ values obtained from both experimental and global-fit data.

The aggregation of insulin was performed under a range of protein concentrations from 0.2 to 1.0 mM in a 20% acetic acid solution, containing no GAO or GA (Fig. 1A), with 200 µM GA (Fig. 1B) and with a range of GAO concentrations (Fig. 1C–F). GA had nearly no visible influence on the aggregation half-times, apart from a small effect on the lowest insulin concentrations (Fig. 1A,B). However, when GA was oxidised, the inhibitory effect became much more potent, with larger t_50_ values seen even at low GAO concentrations (Fig. 1C). Double logarithmic plots of the aggregation half-times versus insulin concentrations are linear under all GAO conditions (Fig. 1A–F inserts), suggesting that the overall aggregation mechanism remains the same and there are no saturation or competition effects present^49^.

**Figure 2:**
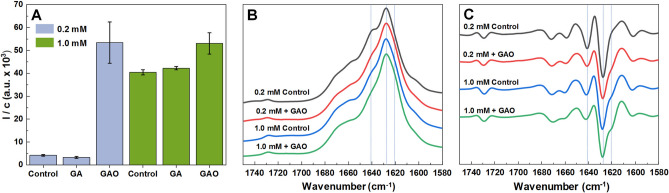
Fluorescence emission intensity and fibril concentration ratios calculated by dividing the sample’s fluorescence intensity by its protein concentration (**A**). FTIR (**B**) and second derivative spectra (**C**) of insulin fibrils formed from 0.2 mM and 1.0 mM insulin with or without 200 µM GAO.

The ThT fluorescence intensity and fibril concentration ratios (sample fluorescence intensity divided by its protein concentration) at the end of the reaction were compared in order to determine if there are any differences (Fig. 2A) when insulin is aggregated in the absence or presence of 200 µM GA or GAO (Fig. S3). GAO leads to a tenfold higher ratio when insulin is aggregated at 0.2 mM concentration, while GA has no effect. In the case of 1.0 mM insulin, the ratio is similar between samples containing no additives or GA and they are only slightly lower when compared to the sample with GAO. In all four cases, the FTIR spectra (Fig. 2B) exhibit a maximum at 1628 cm^−1^ with a shoulder at 1641 cm^−1^ in the amide I/I′ region. Three of the four spectra look nearly identical, with 0.2 mM without GAO being the odd one out with the more pronounced shoulder. It is reflected in the second derivative FTIR spectra (Fig. 2C) in a more pronounced minimum and displays a minor shoulder at 1641 cm^−1^. Moreover, the shoulder at 1620 cm^−1^ is visible in all second order derivative spectra, except the case of 0.2 mM insulin fibrils prepared without GAO. All of this suggests differences in the secondary structure of fibrils. This is in line with the ThT intensity-fibril concentration ratio distribution, which shows that the 0.2 mM sample without GAO is distinct from the rest.

**Figure 3:**
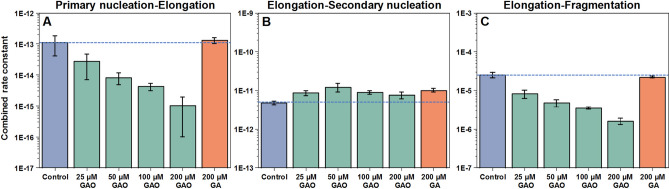
Insulin aggregation primary nucleation-elongation (**A**), elongation-secondary nucleation (**B**) and elongation-fragmentation (**C**) combined rate constants when there is 200 µM GA or different GAO concentrations present in solution. Rate constants were obtained by global-fitting the entire concentration range (0.2–1.0 mM) of insulin aggregation kinetic data at a specific GA or GAO concentration.

Global fitting of the kinetic curves resulting from a range of protein concentrations at different GAO concentrations reveals that there is a sizeable decrease in the combined primary nucleation-elongation rate constant and a considerable decrease in the combined elongation-fragmentation rate constant, while the combined elongation-secondary nucleation constant experiences minimal changes (Fig. 3, Table S1). In some cases, the fit is not ideal at low concentrations due to the stochastic nature of insulin aggregation^50^. Non-oxidised GA had nearly no effect on any of the combined rate constants.

### Seeded aggregation

**Figure 4:**
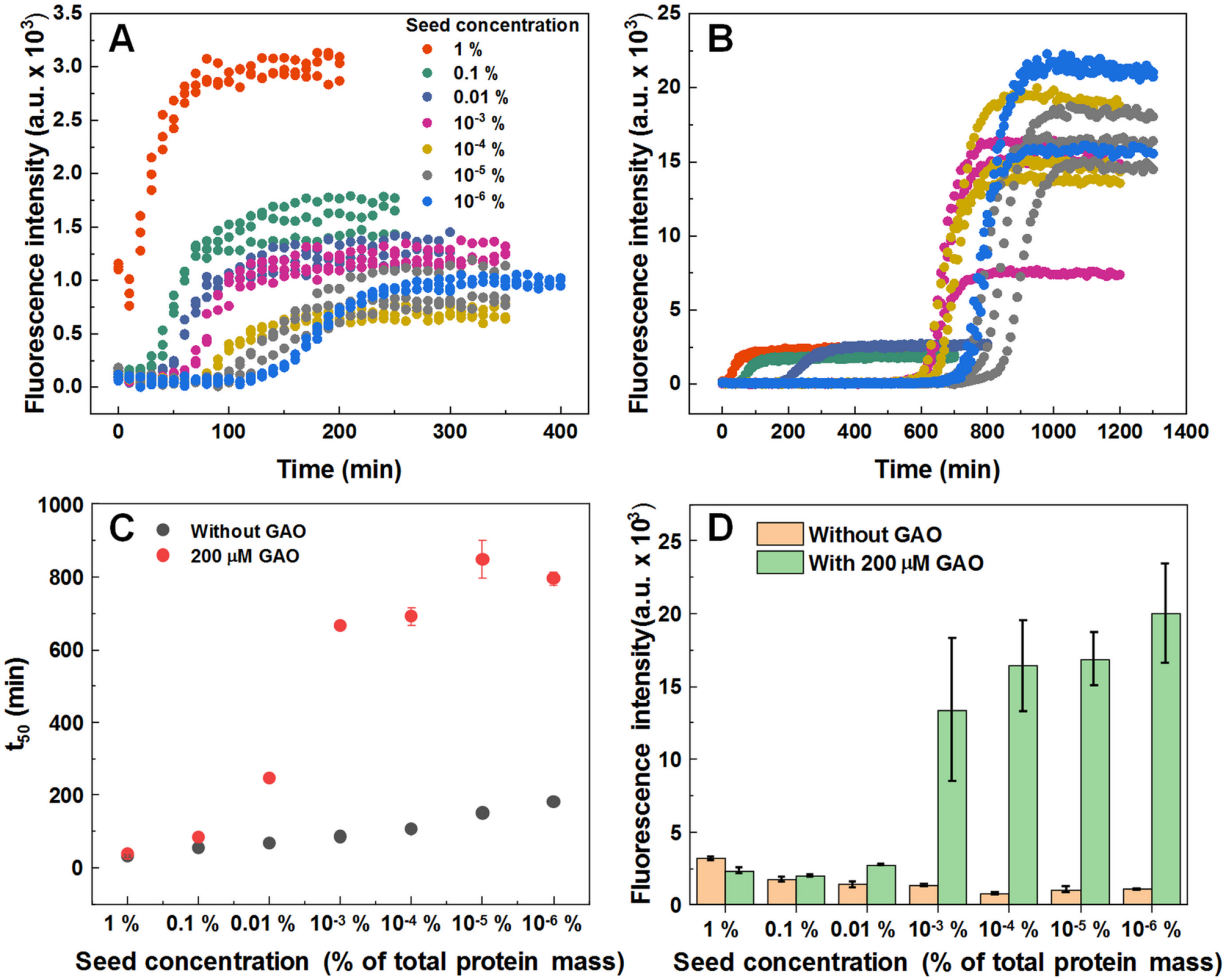
Seeded aggregation kinetic curves of insulin without (**A**) and with (**B**) 200 µM GAO and their t_50_ values (**C**). The fluorescence intensity of fibrils formed during seeded aggregation with and without 200 µM GAO (**D**).

To determine whether GAO has an effect on fibril elongation, seeded aggregation (Fig. 4A,B) was performed using fibrils prepared from a 0.2 mM insulin solution (aggregates that possess a relatively low bound-ThT fluorescence). When the amount of seed present in solution is high, GAO has almost no effect on the reaction t_50_ values (Fig. 4C) or the resulting ThT fluorescence intensity (Fig. 4D). This indicates that the seed replicates its structure via elongation and GAO has virtually no influence on this process. However, when the seed concentration is low (10^−3^–10^−6^%) and nucleation events have a substantial contribution to the aggregation process, GAO causes a significant increase in both the t_50_ values, as well as bound-ThT fluorescence intensity. This higher intensity is attributed to fibrils formed at 1.0 mM insulin concentration or when GAO is present during spontaneous aggregation. This suggests that the presence of GAO affects predominantly nucleation events and induces the formation of a different fibril conformation.

### Fibril morphology

**Figure 5:**
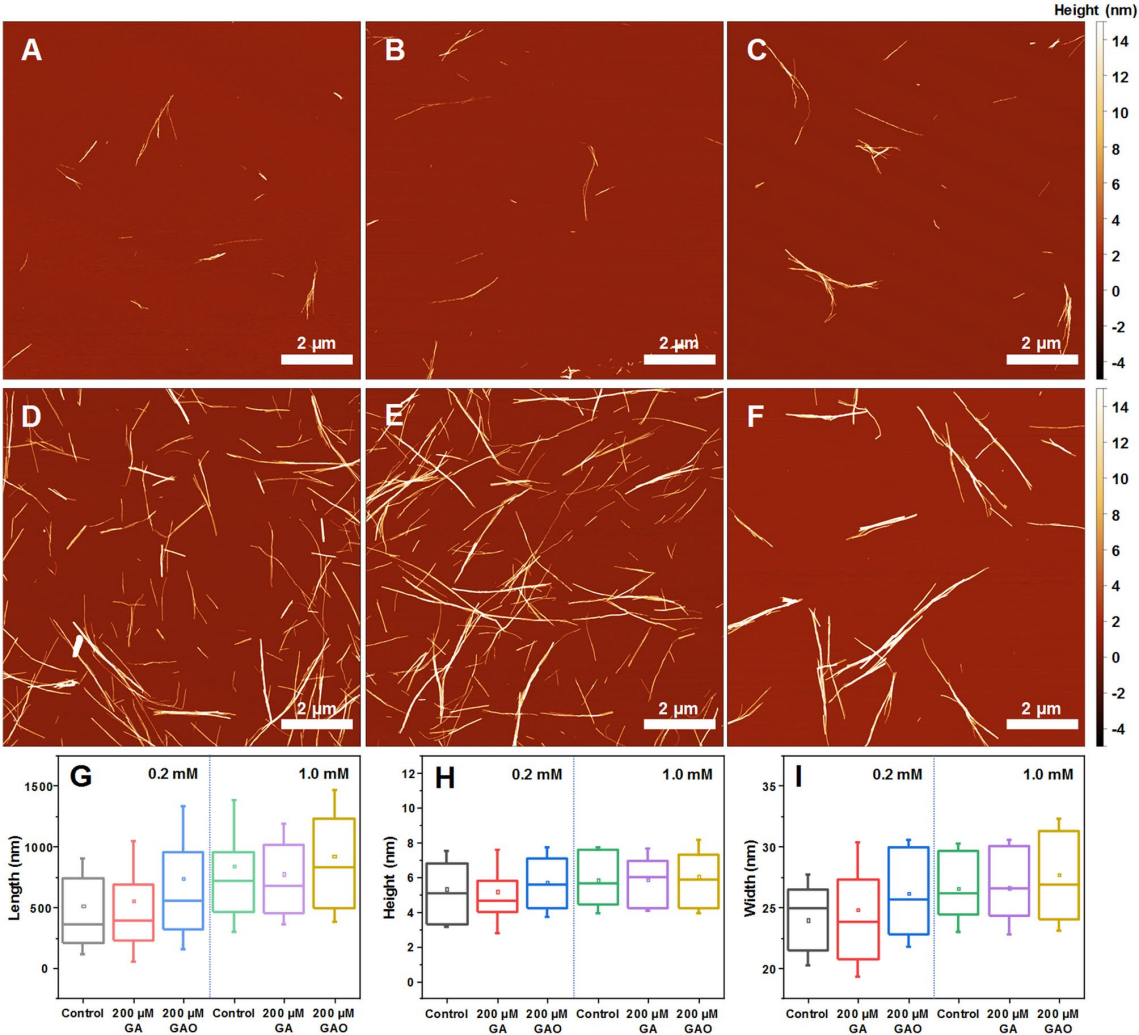
AFM images of insulin fibrils formed without (**A**, **D**) and with 200 µM of GA (**B**, **E**) or GAO (**C**, **F**) at 0.2 mM and 1.0 mM protein concentration respectively. Fibril length (**G**), height (**H**) and width (**I**) distribution, where box plots indicate the interquartile range and errors bars are for 1 standard deviation (n = 50).

The morphology of insulin fibrils formed at different protein and GA or GAO concentrations was compared using AFM (Fig. 5). In the case of 0.2 mM insulin without GAO (Fig. 5A and Fig. S4) or with 200 µM GA (Fig. 5B and Fig. S4), the fibrils are mostly dispersed and short. When there is 200 µM GAO present in the sample (Fig. 5C and Fig. S4), the formed aggregates are longer (Fig. 5G), wider (Fig. 5I) and more prone to self-association, while their average height (Fig. 5H) remains relatively even. In the case of 1.0 mM insulin, all conditions lead to longer fibrils that are prone to self-association (Fig. 5D–F and Fig. S5). There is also a slight increase in their average length (Fig. 5G and Fig. S6). While in both cases length and width experience a GAO concentration-dependent change, there appears to be almost no effect on fibril height (Fig. S6). These GAO-induced changes in morphology further support the hypothesis that GAO alters the pathway of fibril formation, which is especially visible in the case of 0.2 mM insulin.

## Discussion

The unseeded aggregation kinetic data shows that at low pH values, GA has virtually no effect on insulin aggregation, while GAO inhibits it quite effectively (Fig. 1). This could be the reason why experiments conducted at neutral pH, where GA can undergo oxidation, show an effective inhibition of amyloid formation. Due to this factor, the inhibitory effect can be best analysed using pre-oxidised gallic acid at low pH values, where it no longer experiences any further oxidation.

Fitting the spontaneous aggregation kinetic data with the “classic” aggregation model reveals that GAO most effectively inhibits primary nucleation (Fig. 3A), as 200 µM GAO results in a 100-fold decrease in the rate of nuclei formation. There is also a considerable decrease in fragmentation rates (Fig. 3C). Despite the massive effect on primary nuclei formation, secondary nucleation appears to be almost unaffected, even at the highest GAO concentrations (Fig. 3B).

**Figure 6:**
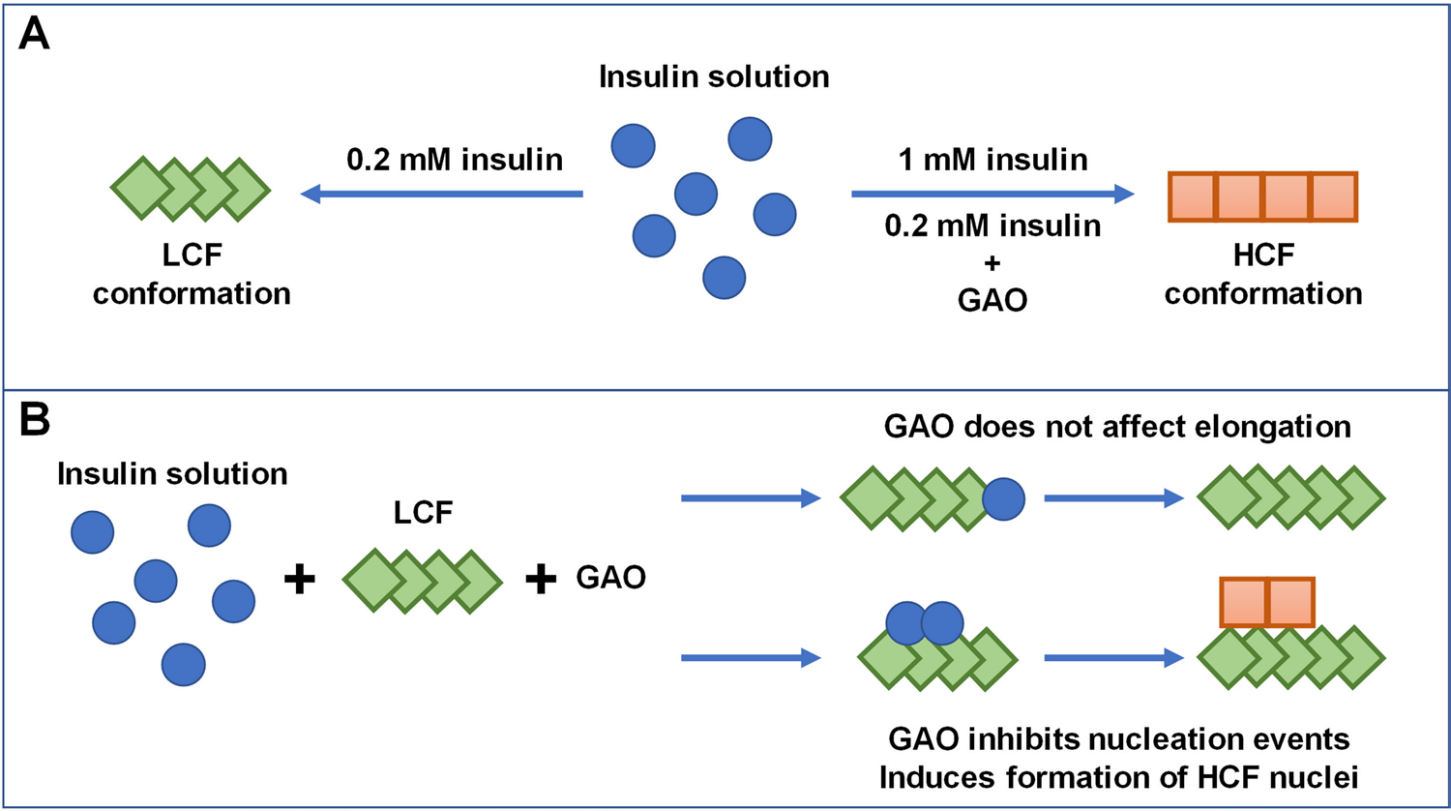
Formation of distinct insulin fibril conformations based on protein concentration and the absence or presence of GAO (**A**). Effect of GAO on LCF elongation, as well as nucleation events.

A previous report demonstrated that distinct fibril conformations can be formed at 0.2 mM and 1 mM insulin concentrations, termed LCF (low concentration fibrils) and HCF (high concentration fibrils), respectively^46^. When 0.2 mM insulin aggregates in the presence of GAO, the fibril-bound-ThT fluorescence intensity is much higher than typically observed for the LCF conformation, and the intensity-concentration ratio is similar to the HCF conformation (Fig. 2A). It is likely that GAO induces formation of HCF, which is further supported by the differences in secondary structure, as examined by FTIR (Fig. 2B,C), where fibrils formed with GAO possess a similar secondary structure as HCF (Fig. 6A). Interestingly, even the HCF sample ratio experiences a slight increase with the addition of GAO, suggesting that without GAO it likely contains some LCF as well.

Seeded aggregation data shows that at high initial LCF concentrations, GAO has virtually no effect on the t_50_ value (Fig. 4A–C), indicating that it does not affect the rate of elongation (Fig. 6B). At high concentrations, the LCF seed replicates its conformation and we observe no differences in ThT fluorescence intensity between samples with and without GAO (Fig. 4D). However, once the amount of seed reaches a low enough value, there is a drastic change in t_50_ values and ThT fluorescence intensities between both conditions, indicating that GAO causes the formation of HCF.

When GAO is present, the dependence of t_50_ on initial seed concentration is not linear on a logarithmic scale. This suggests that the process is more complex and involves not just inhibition of primary nucleation. Since the concentration of seed appears to factor into this effect, it is possible that GAO affects the rate of secondary nucleation on the surface of fibrils (Fig. 6B).However, fitting the data of spontaneous aggregation does not display any significant differences in the combined elongation-secondary nucleation rate constants (Fig. 3B). Considering that the fit model is comprised of four basic aggregation steps, it would be unable to account for any additional processes or inhibitor-fibril interactions. One possible explanation for this effect is that GAO does inhibit secondary nucleation by hiding the accessible surface of fibrils, but its affinity towards the fibril’s surface is so low that only a small amount of fibrils can be effectively covered. When more aggregates are present, it is simply incapable of effectively covering the fibril surface area. Seeing as its effect on secondary nucleation diminishes at around 0.01% fibril concentration (Fig. 4C), such a tiny amount of aggregates would likely not be detected in the spontaneous aggregation experiments, which is why fitting their kinetic data shows virtually no effect on secondary nucleation.

If we consider the effect of GAO based on the spontaneous and seeded aggregation kinetic data, it seems that it is potent at inhibiting the formation of nuclei and has no influence on the elongation process. Based on the differences in bound-ThT fluorescence data, as well as AFM images and FTIR spectra, it appears that GAO is also capable of altering the aggregation pathway of these nuclei. Even under conditions which would favour the formation of the LCF conformation, GAO manages to alter the fibrillization process towards HCF. The non-oxidised form of GA, on the other hand, has no visible effect on the aggregation kinetics, nor does it change the resulting fibril type.

## Conclusions

Gallic acid does not affect the aggregation of insulin at low pH and it only gains its inhibitory potential after undergoing oxidation. The oxidized form is highly effective at inhibiting primary nuclei formation, while having no effect on fibril elongation. It also appears to alter the formation pathway of insulin amyloid aggregation, resulting in HCF even at low protein concentrations.

## Supplementary information

Supplementary file1

## Data Availability

The datasets generated and/or analysed during the current study are available from the corresponding author on reasonable request.
