# Supplementary material for: Gallic acid oxidation products alter the formation pathway of insulin amyloid fibrils
Source: Sci Rep. 2020 Sep 2;10:14466. doi: 10.1038/s41598-020-70982-3 (PMC7468289; doi:10.1038/s41598-020-70982-3)
Supplement: Supplementary file 1 — Supplementary file1 [file 41598_2020_70982_MOESM1_ESM.pdf]

Gallic Acid Oxidation Products Alter the Formation Pathway of Insulin Amyloid Fibrils

Andrius Sakalauskas, Mantas Ziaunys, Vytautas Smirnovas

Institute of Biotechnology, Life Sciences Center, Vilnius University, Vilnius, Lithuania

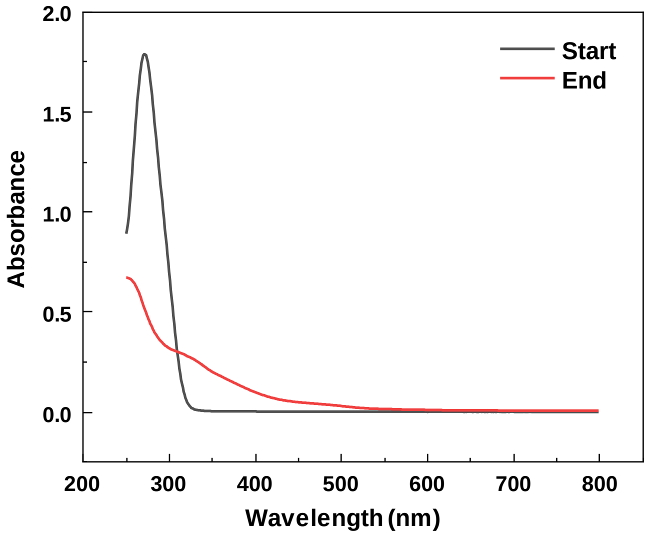

Supplementary Figure S1. Gallic acid solution absorbance at the start and the end of the oxidation reaction, measured between 250 nm and 800 nm using a Shimadzu UV-1800 spectrophotometer. The gallic acid solution was diluted to 200 µM before measurement. Each spectrum is the average of 3 measurements.

Supplementary Table S1. Insulin aggregation combined rate constants at 200 µM GA and different GAO concentrations.

	**1° nucleation - elongation**	**Elongation- 2° nucleation**	**Elongation- fragmentation**
**Control**	**$(1.1 \pm 0.7) \times 10^{-13}$**	**$(4.7 \pm 0.5) \times 10^{-12}$**	**$(2.5 \pm 0.4) \times 10^{-5}$**
**200 µM GA**	**$(1.3 \pm 0.3) \times 10^{-13}$**	**$(1.0 \pm 0.1) \times 10^{-11}$**	**$(2.2 \pm 0.1) \times 10^{-5}$**
**25 µM GAO**	**$(2.7 \pm 2.0) \times 10^{-14}$**	**$(8.5 \pm 1.3) \times 10^{-12}$**	**$(8.1 \pm 1.9) \times 10^{-6}$**
**50 µM GAO**	**$(8.1 \pm 3.4) \times 10^{-15}$**	**$(1.2 \pm 0.3) \times 10^{-11}$**	**$(4.7 \pm 1.0) \times 10^{-6}$**
**100 µM GAO**	**$(4.2 \pm 1.1) \times 10^{-15}$**	**$(8.8 \pm 0.9) \times 10^{-12}$**	**$(3.5 \pm 0.2) \times 10^{-6}$**
**200 µM GAO**	**$(1.0 \pm 0.9) \times 10^{-15}$**	**$(7.5 \pm 1.5) \times 10^{-12}$**	**$(1.6 \pm 0.3) \times 10^{-6}$**

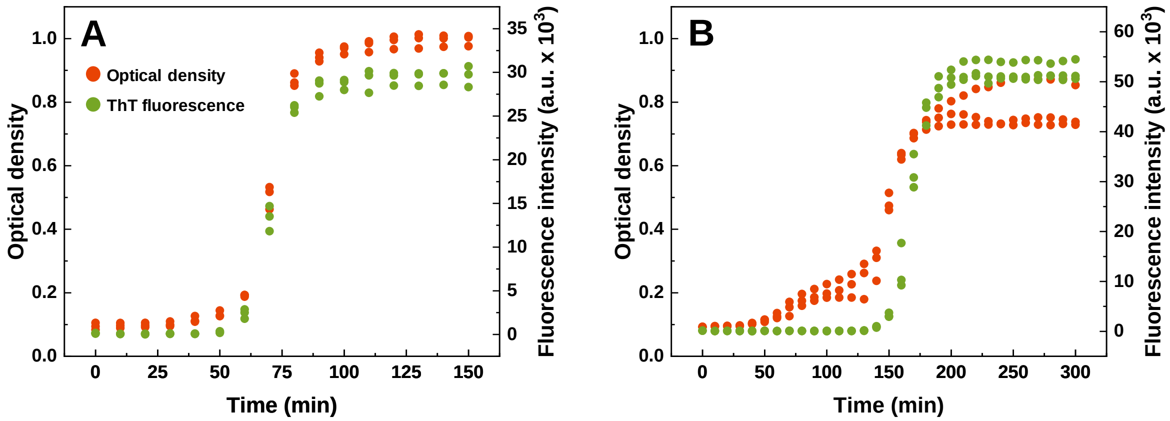

Supplementary Figure S2. Comparison between aggregation kinetics tracked by measuring the solution's optical density and ThT fluorescence intensity of 1.0 mM insulin in the absence (A) or presence (B) of 200 µM GAO.

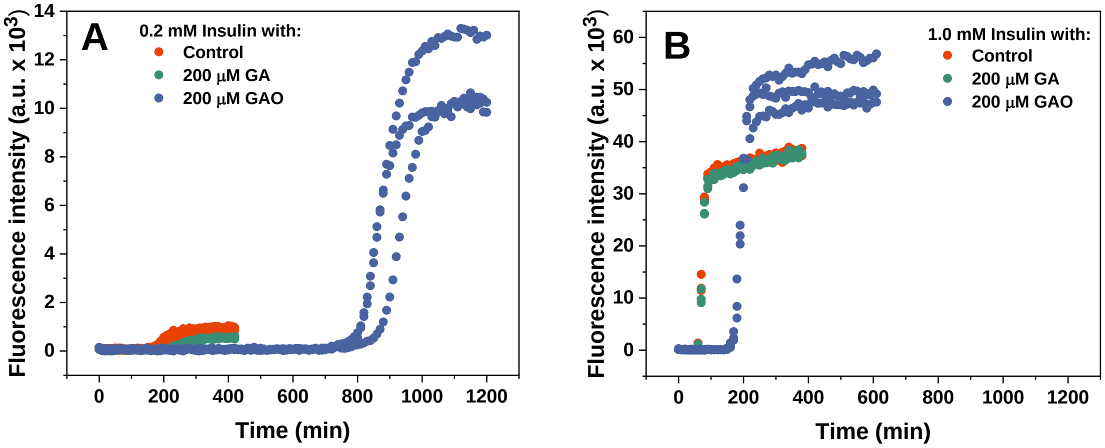

Supplementary Figure S3. Aggregation kinetics of 0.2 mM (A) and 1.0 mM (B) insulin in the absence or presence of 200 µM GA or GAO.

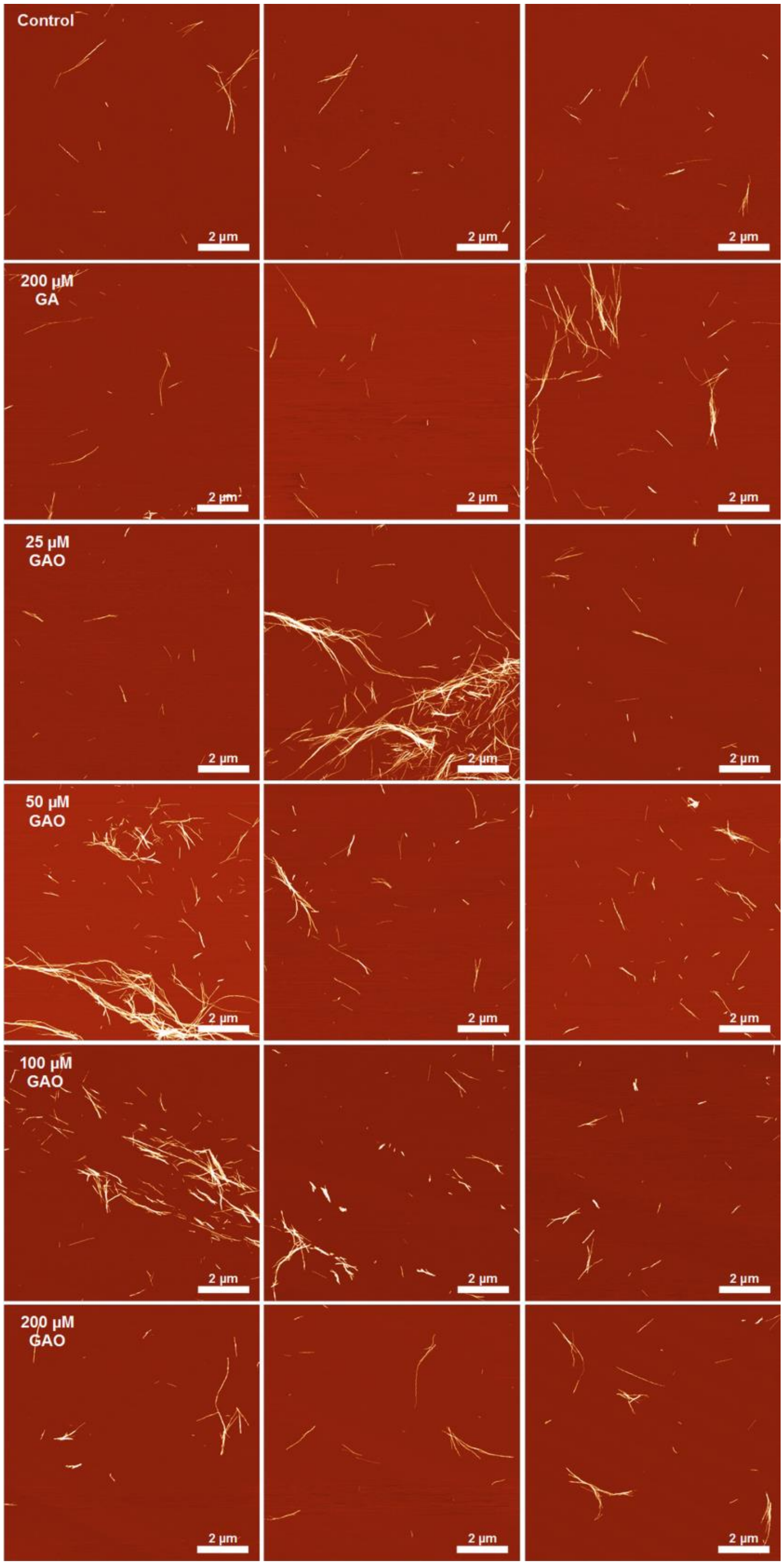

Supplementary Figure S4. AFM images of insulin (0.2 mM) aggregated in the absence and presence of 200 µM GA, 25 µM GAO, 50 µM GAO, 100 µM GAO or 200 µM GAO.

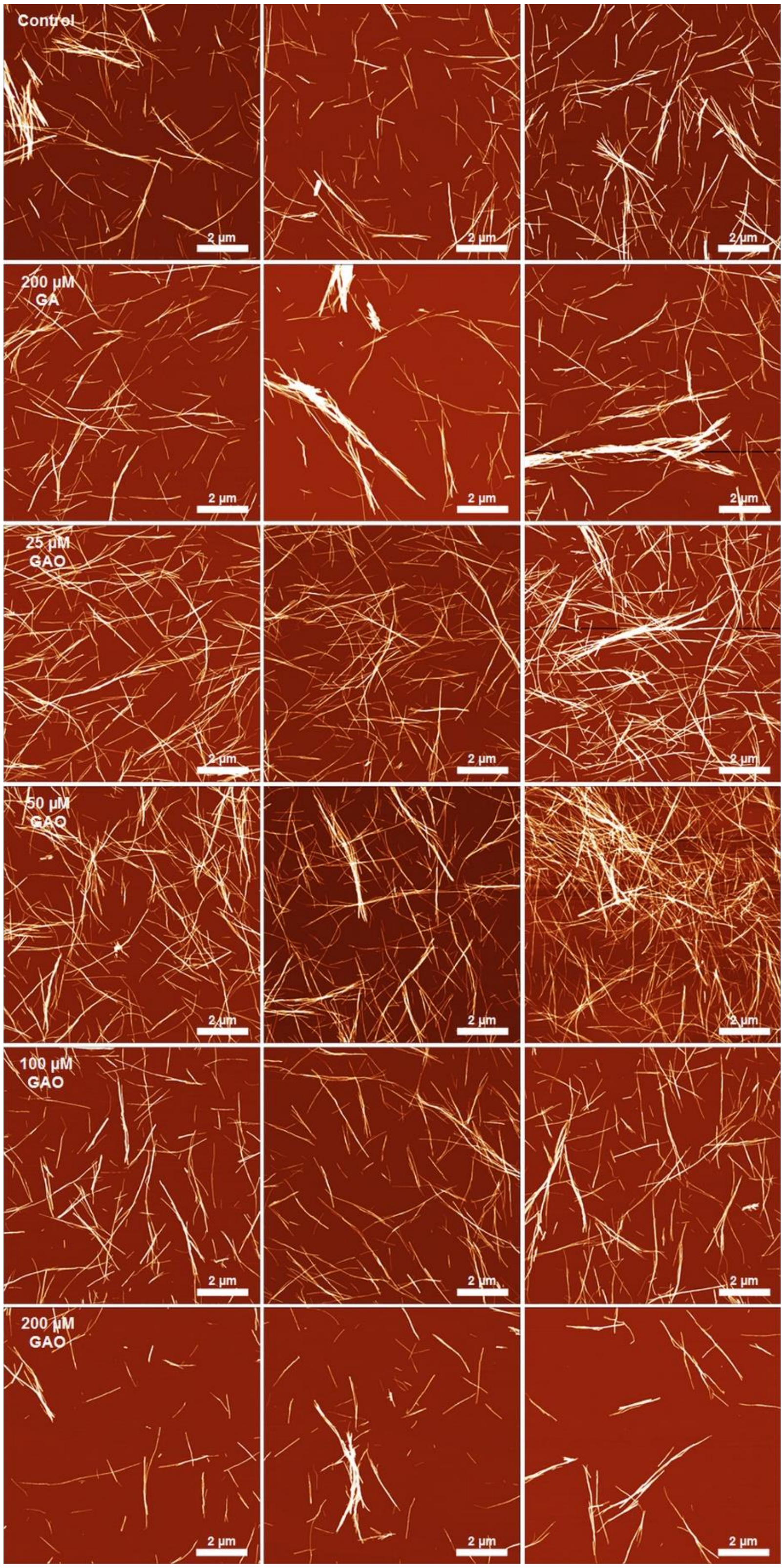

Supplementary Figure S5. AFM images of insulin (1.0 mM) aggregated in the absence and presence of 200 µM GA, 25 µM GAO, 50 µM GAO, 100 µM GAO or 200 µM GAO.

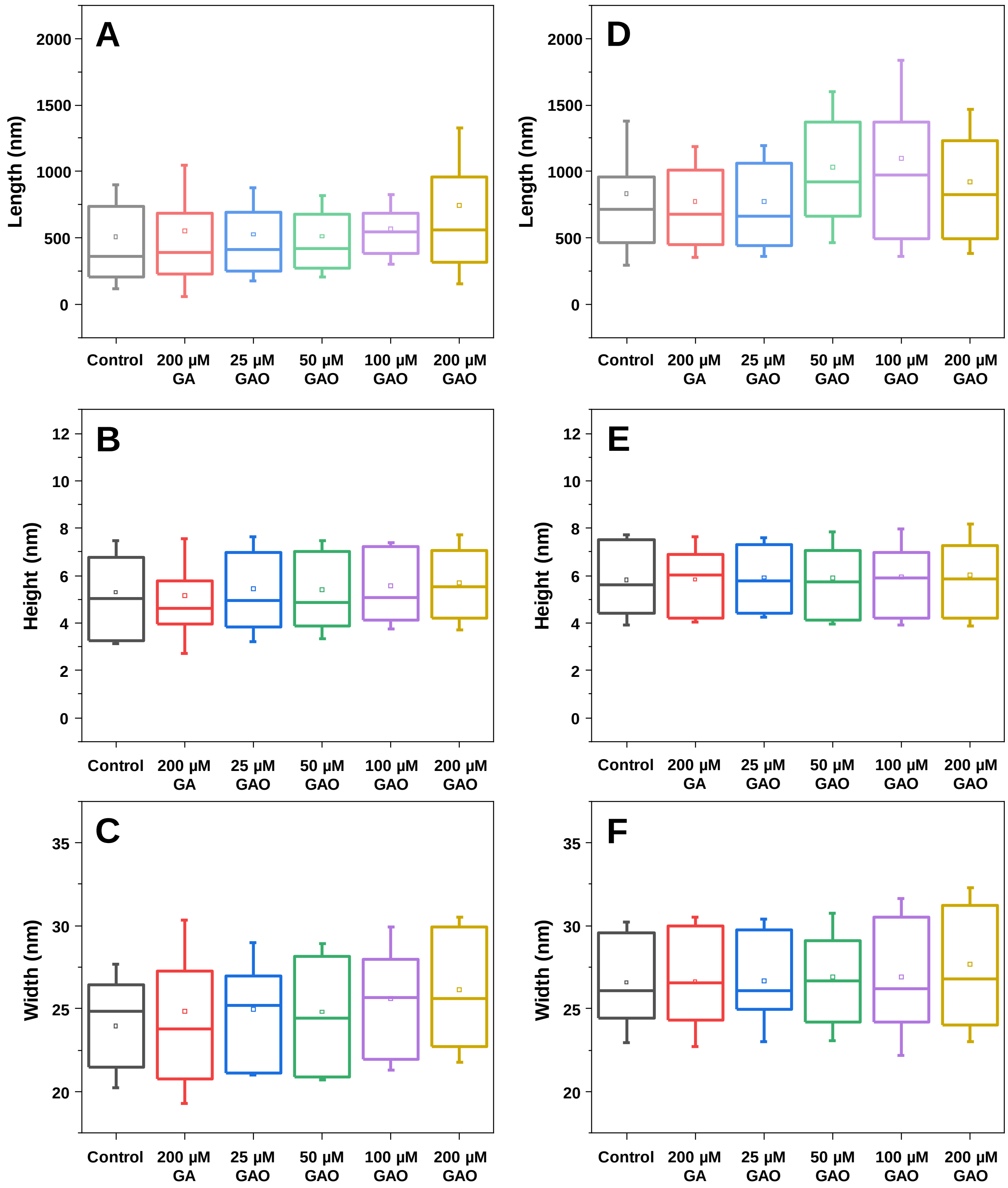

Supplementary Figure S6. Length (A, D), height (B, E) and width (C, F) distribution of insulin fibrils prepared at 0.2 mM and 1.0 mM protein concentrations respectively in the absence and presence of 200 μM GA, 25 μM GAO, 50 μM GAO, 100 μM GAO or 200 μM GAO.

Mathematical framework of data fitting

Fibril concentration was estimated by matching maximum ThT fluorescence intensity to the respective initial insulin concentration for each experimental curve (assuming that aggregation efficiency is 100%) using the following equation:

$$Fibril\ concentration = \frac{(y_x - y_{min}) \times c}{(y_{max} - y_{min})} \tag{1}$$

Where y_x is the ThT fluorescence intensity at time x, y_{min} and y_{max} are the minimal and maximal fluorescence intensities, c – initial insulin concentration.

Primary nucleation, a process in which native protein molecules (M) change their secondary structure and become aggregation centers (A) with rate constant k_n (nucleus size is set to 2):

$$\frac{d[A]}{dt} = k_n[M]^2 \tag{2}$$

Elongation, responsible for fibril growth (F), during which amyloidogenic proteins are added to aggregation centers (A) with rate constant k_+:

$$\frac{d[F]}{dt} = k_+[M][A] \tag{3}$$

Secondary nucleation, the process of aggregation center (A) formation on the surface of fibrils (F) with rate constant k_2, using the surface as a catalyst (nucleus size is set to 2):

$$\frac{d[A]}{dt} = k_2[M]^2[F] \tag{4}$$

Fibril fragmentation, resulting in the creation of new aggregation centers (A) due to breaks in the fibrils (F) with rate constant k_-:

$$\frac{d[A]}{dt} = k_-[F] \tag{5}$$
